# Supplementary material for: Impacts of Zika emergence in Latin America on endemic dengue transmission
Source: Nat Commun. 2019 Dec 16;10:5730. doi: 10.1038/s41467-019-13628-x (PMC6915707; doi:10.1038/s41467-019-13628-x)
Supplement: Supplementary file 1 — Supplementary Information [file 41467_2019_13628_MOESM1_ESM.pdf]

Supplementary Information

Impacts of Zika emergence in Latin America on endemic dengue transmission

Borchering et al.

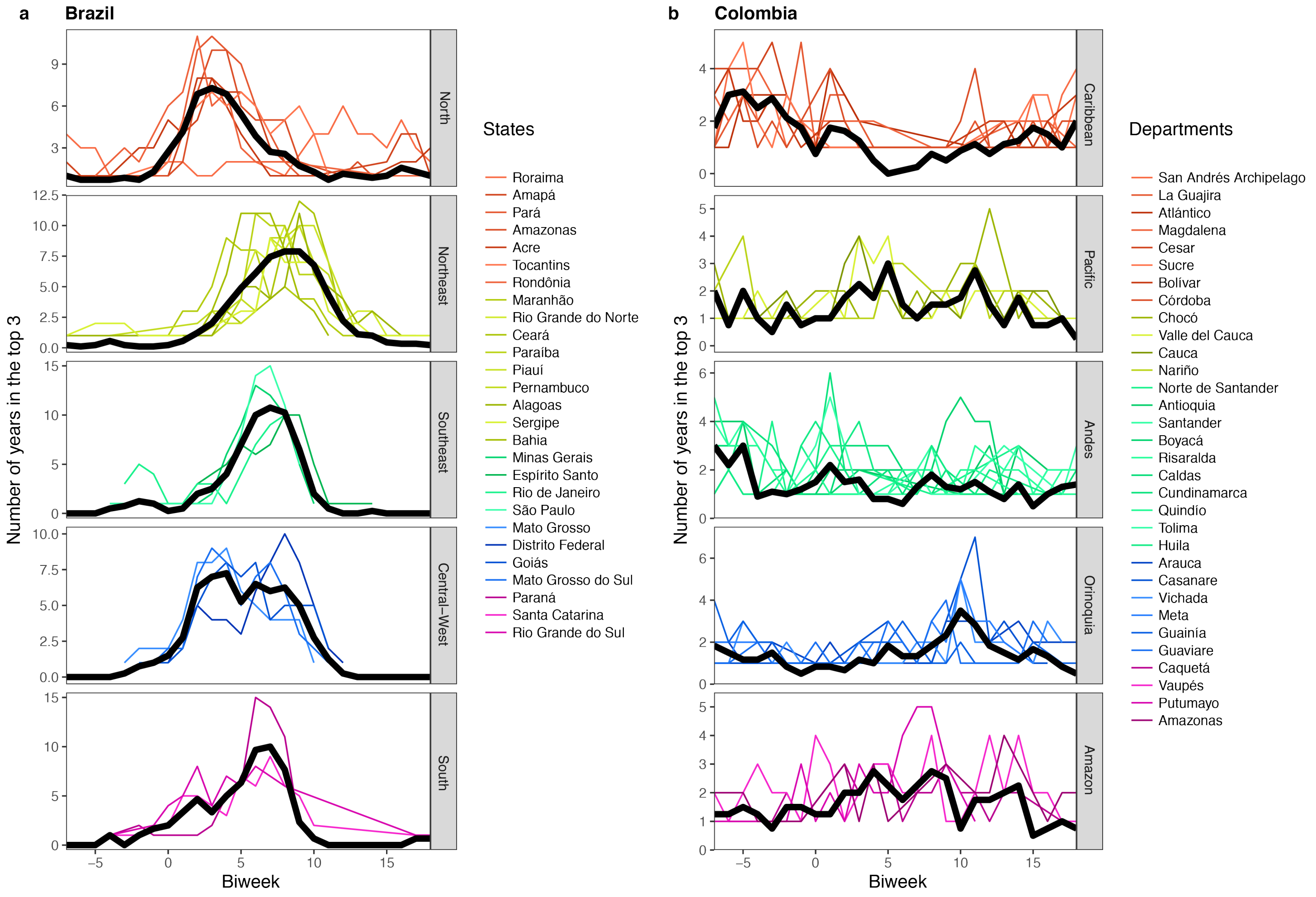

Supplementary Figure 1: Dengue seasonality by biweeks with the top three highest incidence values for each season. Seasons are defined to range from biweek 18 to biweek 17 of the following year (biweek 26 is represented as biweek 0 below). Frequencies of peak biweek are displayed for each state in Brazil (**a**) and department in Colombia (**b**). Black lines show the average frequency for each biweek across locations within each region.

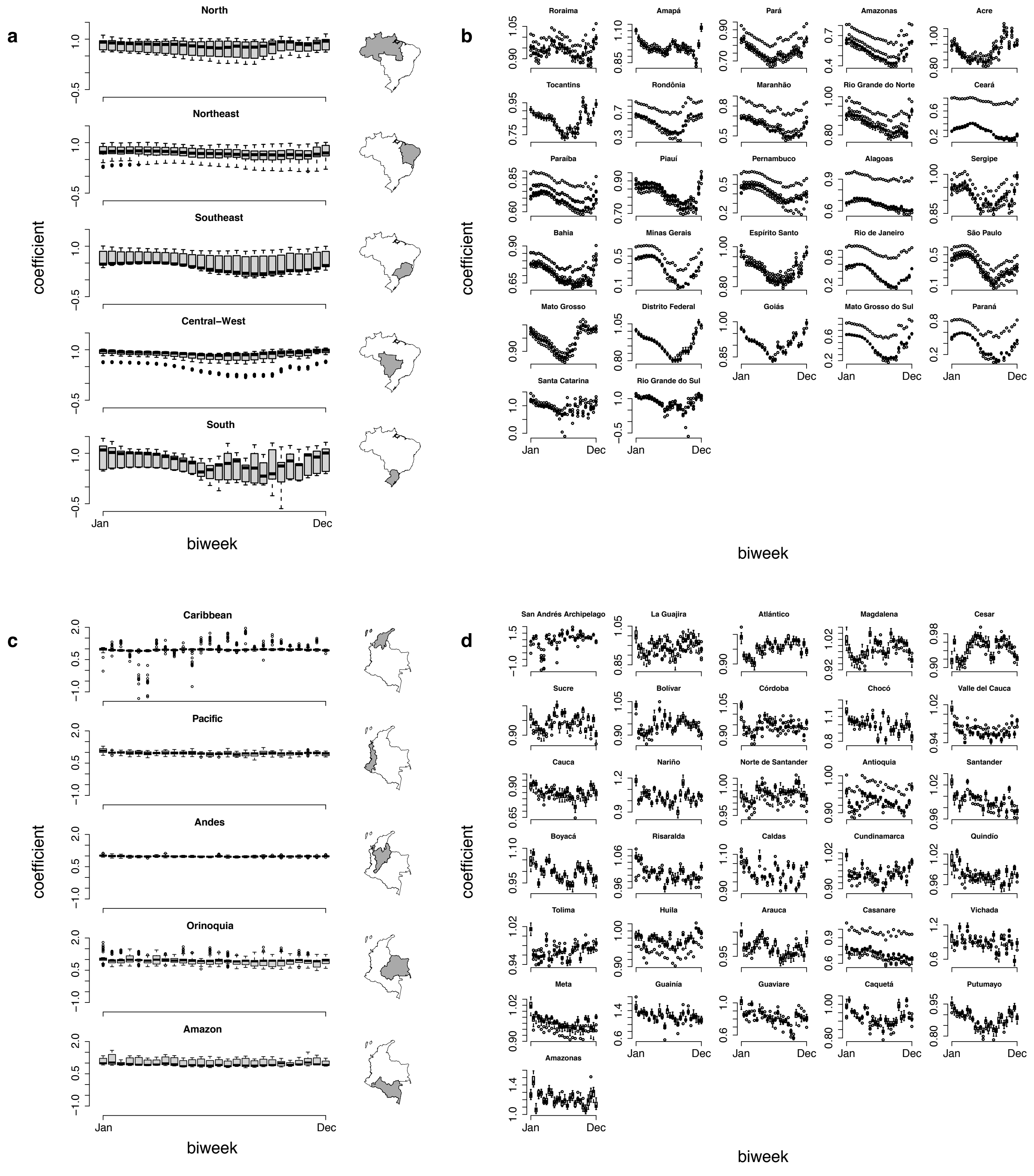

Supplementary Figure 2: Variation in the stan model coefficients for each biweek. Results are shown for coefficients aggregated at the region (**a, c**) and state (Brazil in **b**) or department (Colombia in **d**) level.

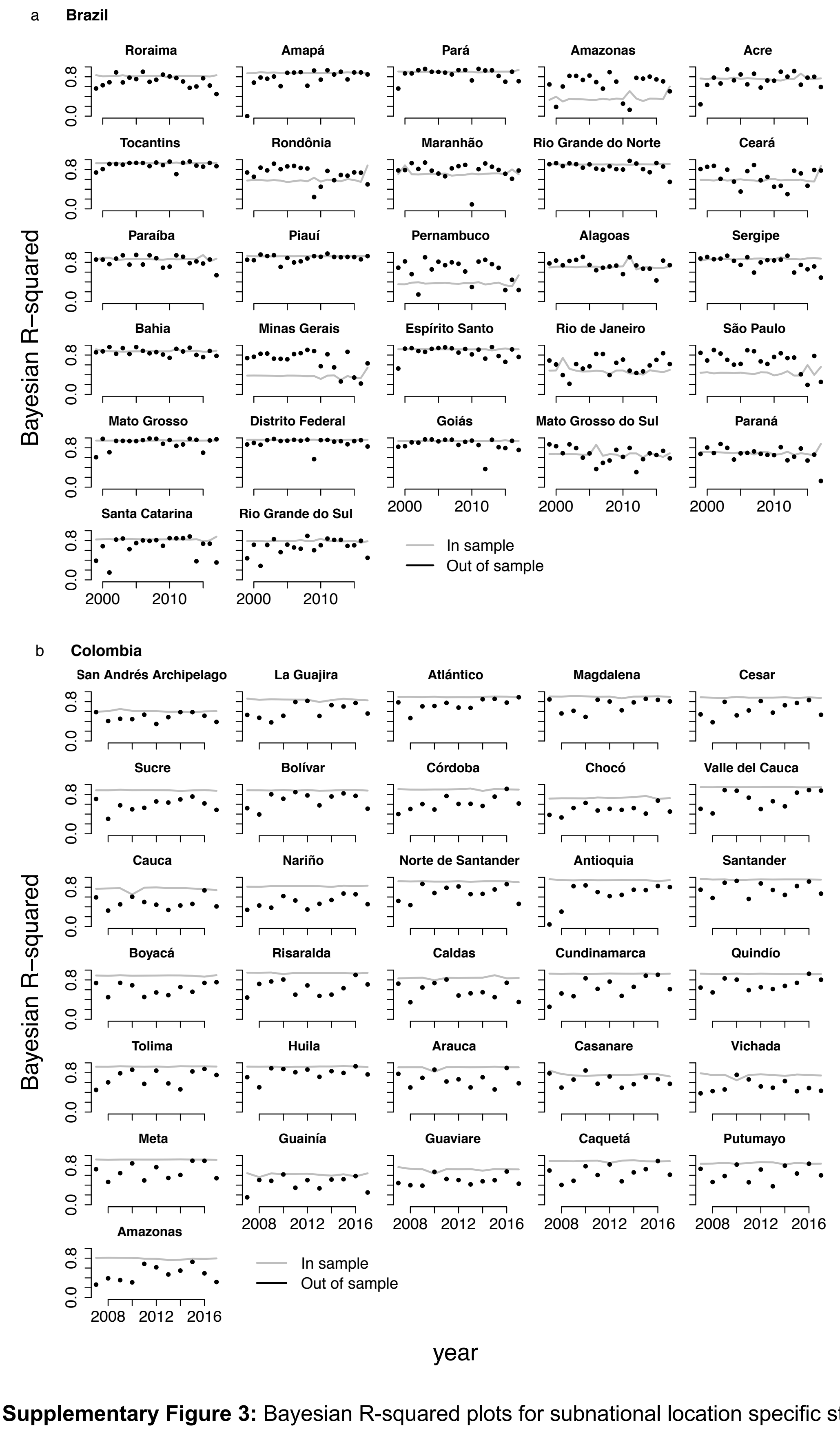

Supplementary Figure 3: Bayesian R-squared plots for subnational location specific stan model predictions versus observed incidence. Results shown for states in Brazil (**a**) and departments in Colombia (**b**). The gray line corresponds the R-squared value for all of the biweeks that were included in the model fitting. The black dots show the Bayesian R-squared value for the year when it was left out of the model fitting. The x-axis indicates the year that was left out, and ranges over all years in the dataset 1999-2017 in Brazil and 2007-2017 in Colombia.

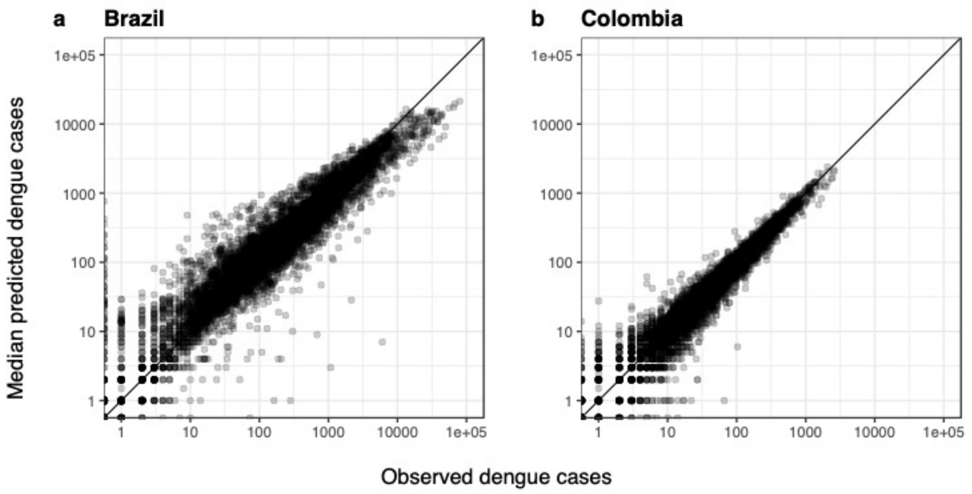

Supplementary Figure 4: Comparison between biweekly predicted and observed dengue incidence for subnational location specific stan models. Each point indicates an observed biweek of dengue incidence for one subnational location and the median value of the corresponding prediction interval. Results are displayed for 2000-2014 in Brazil (**a**) and 2007-2014 in Colombia (**b**) in order to evaluate model performance prior to Zika virus emergence.

a Brazil

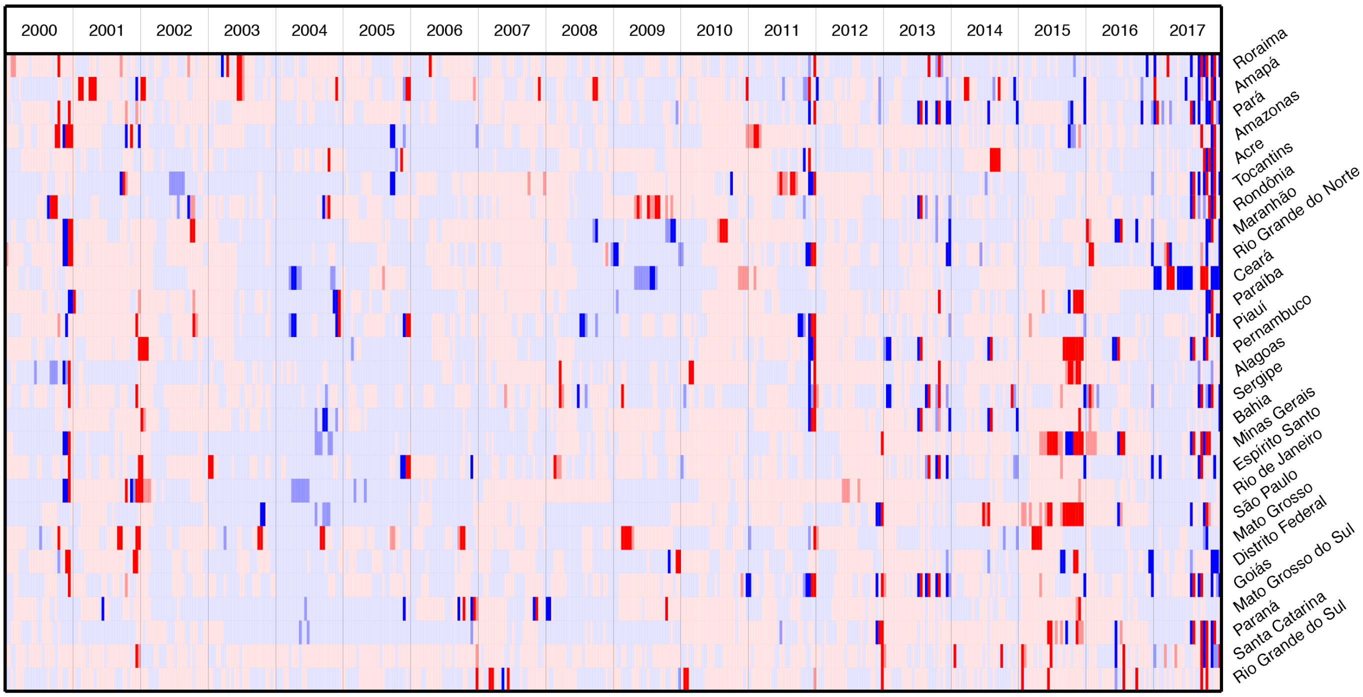

b Colombia

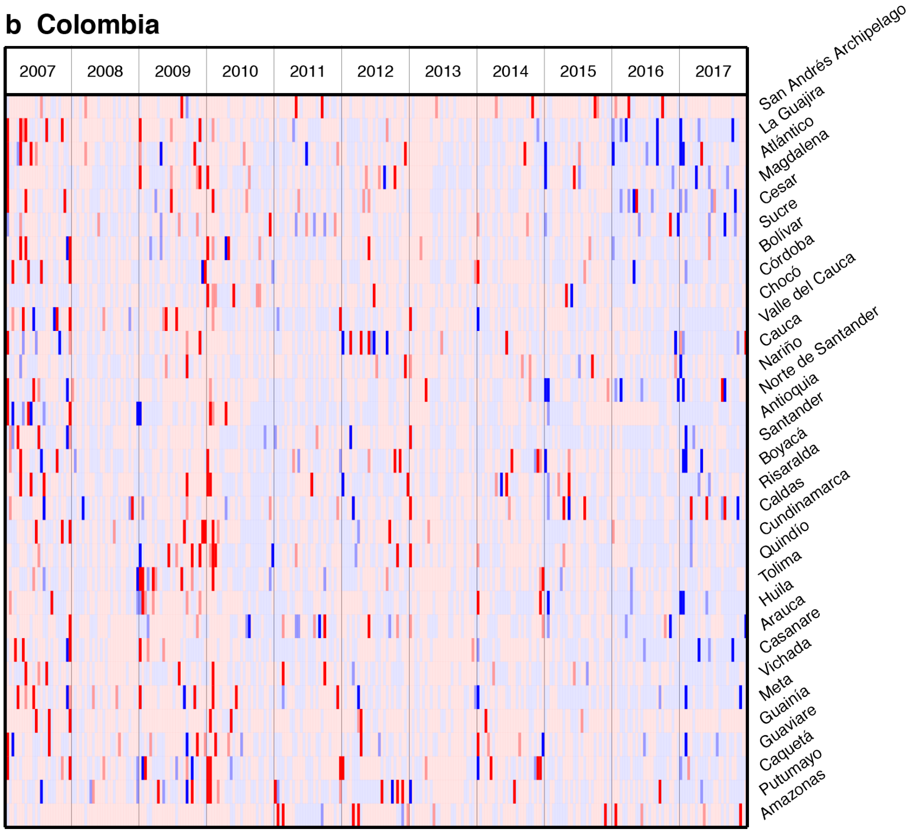

Supplementary Figure 5: Comparison between predicted and observed incidence. Red and blue indicate that the observed incidence was above and below the median value of the posterior distribution of predicted values for that biweek, respectively. Dark biweeks indicate that the observed incidence was outside of the 95% prediction interval and medium shaded biweeks indicate that the observed incidence was outside of the 90% prediction interval. Results are shown for Brazil (**a**) and Colombia (**b**).

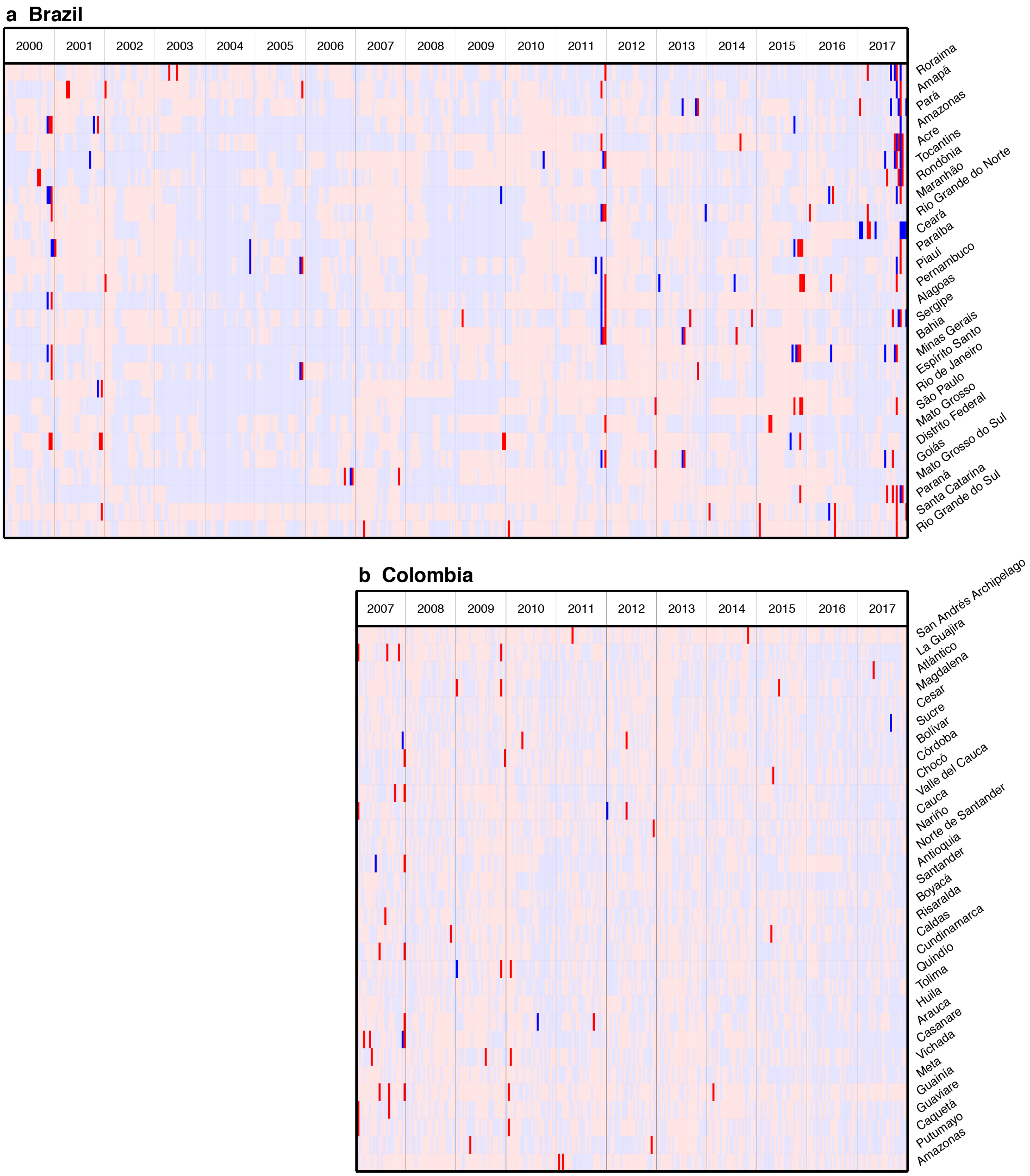

Supplementary Figure 6: Bonferroni adjusted quantile plot for full time series and recent years. Quantile values are based on the location of the observed incidence in the cumulative distribution of 500 sampled posterior prediction values. Results are shown for Brazil (**a**) and Colombia (**b**).

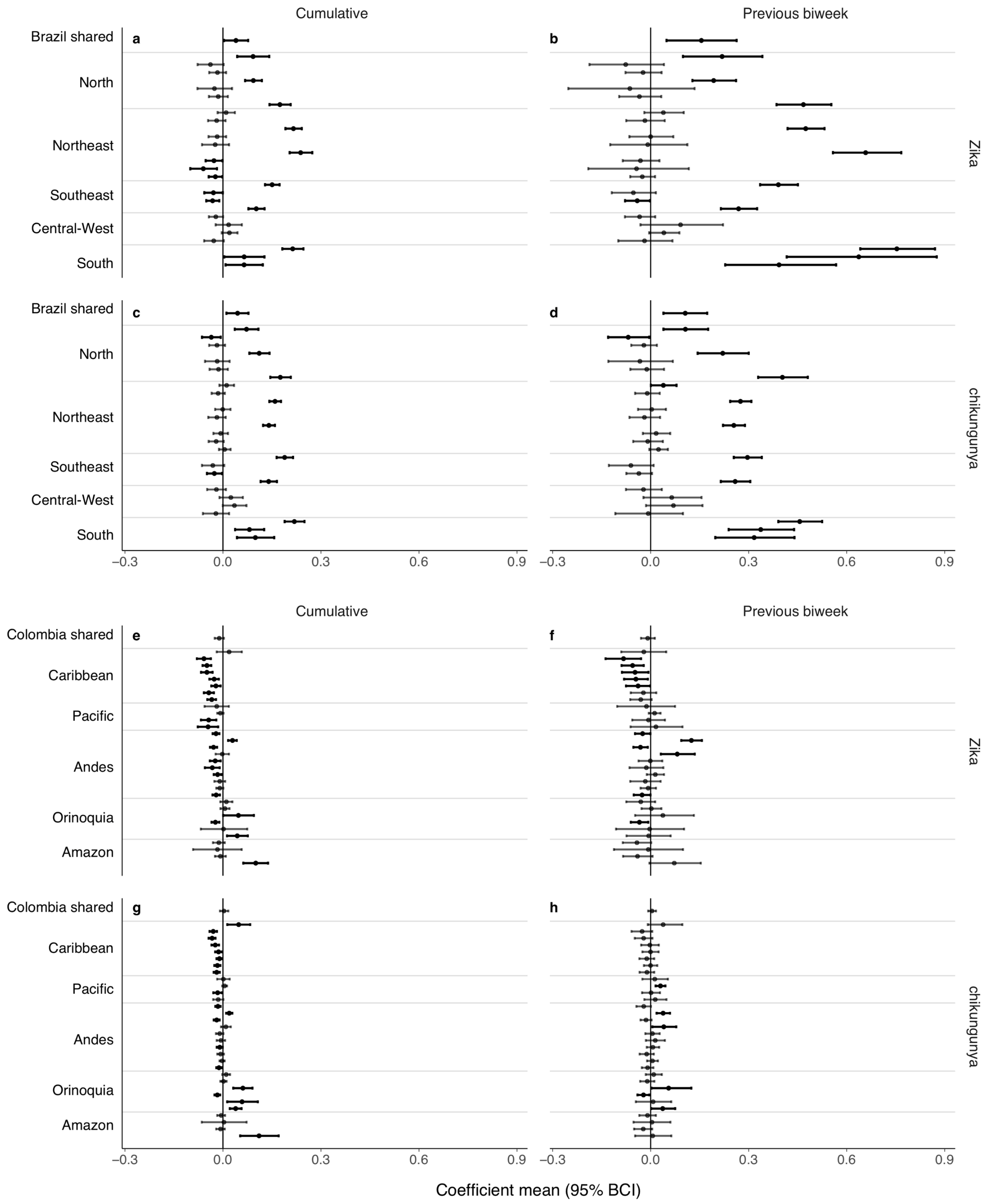

Supplementary Figure 7: Dengue time series model results with spatial hierarchical structure and arboviral disease covariates. Mean and 95% Bayesian credible intervals are displayed for the shared effect (top row) and for the location specific effects (other rows ordered by region and then latitude) for Brazil (**a-d**) and Colombia (**e-h**). Location specific effects are displayed as the sum of shared coefficient and location specific coefficient. Models are displayed for biweekly Zika cases (**b**, **f**), biweekly chikungunya cases (**d**, **h**), cumulative Zika cases (**a, e**), and cumulative chikungunya cases (**c, g**).

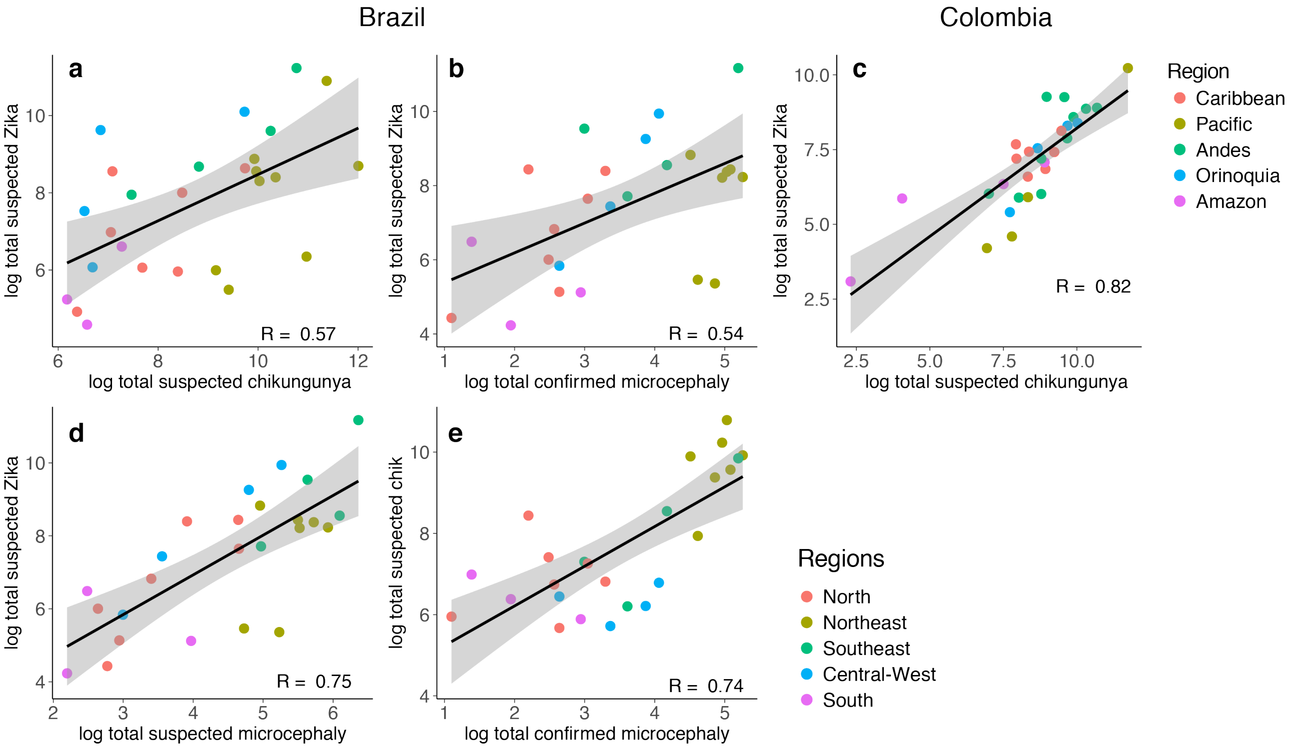

Supplementary Figure 8: Correlation between arbovirus related case count totals. Results are shown for states in Brazil (**a, b, d, e**) and departments in Colombia (**c**). Correlation coefficients are displayed in the bottom right corner of each panel.

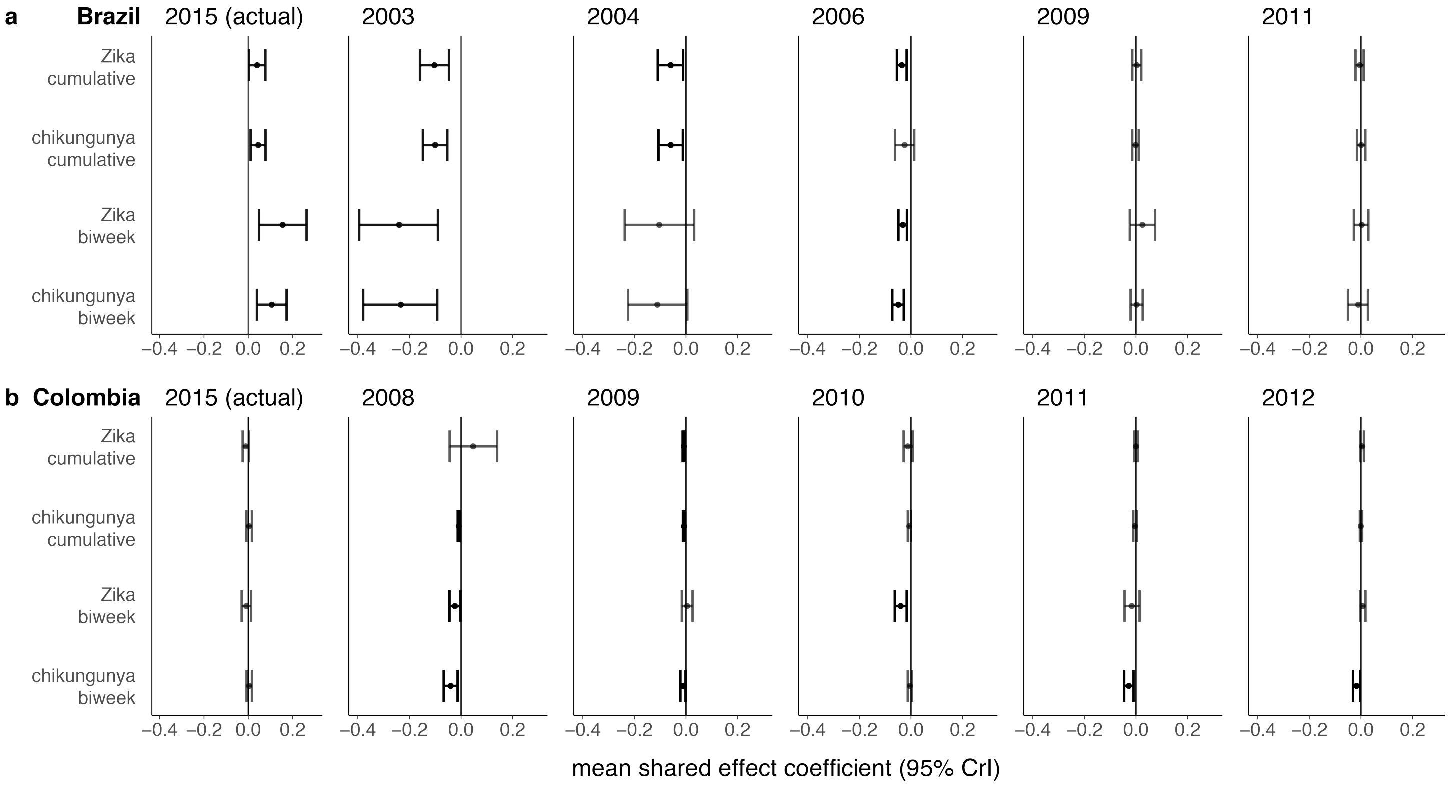

Supplementary Figure 9: Hierarchical model shared effects for permuted recent year datasets. Mean and 95% Bayesian credible intervals are displayed for shared effect coefficients from models fit using the actual dataset (leftmost panels) and alternative datasets where 2015 to 2017 were replaced with three consecutive years of data preceding 2015 for Brazil (**a**) and Colombia (**b**). Nonsignificant results are displayed with light shading.

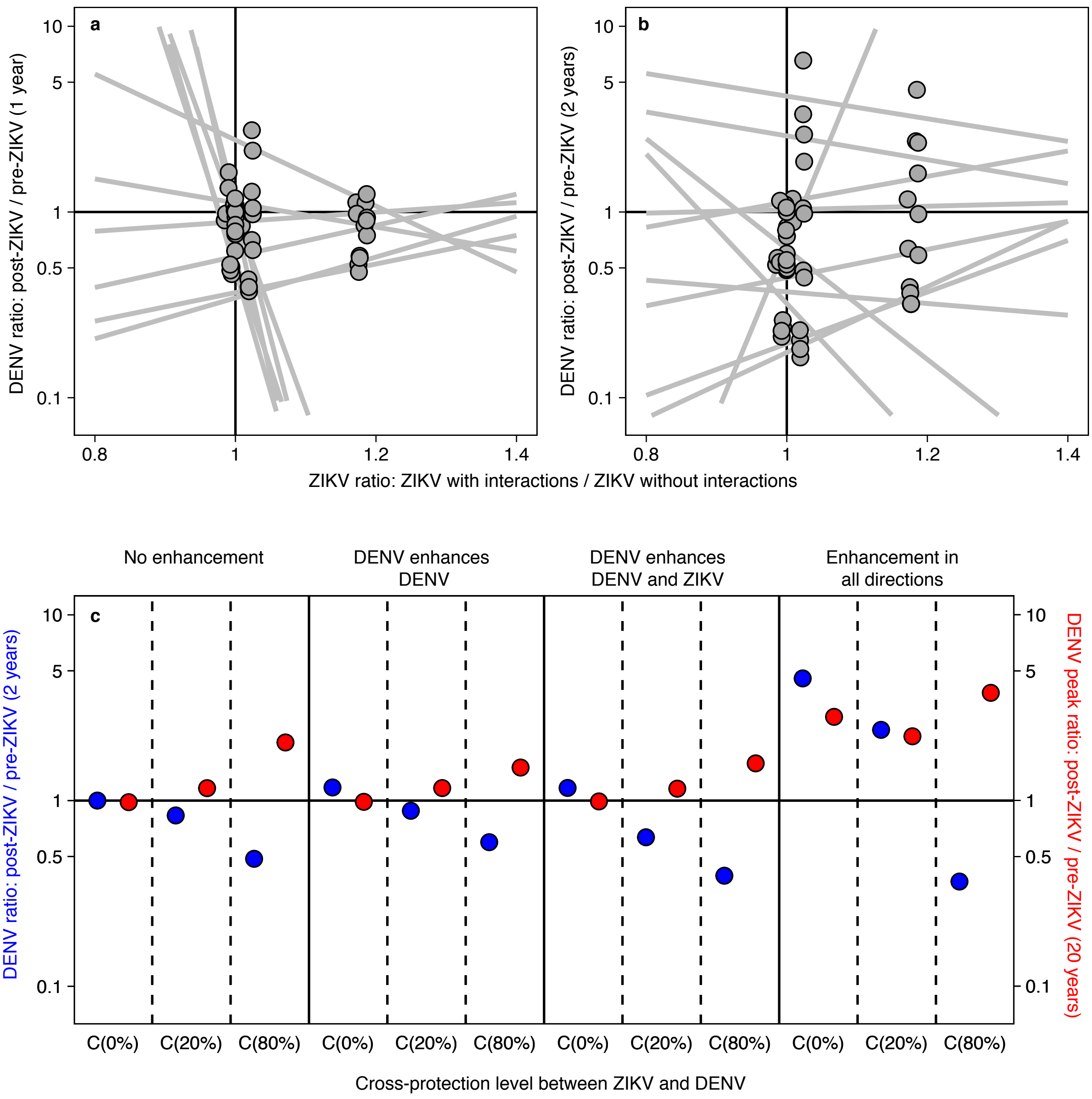

Supplementary Figure 10: Effects of immune-mediated interactions between DENV and ZIKV on case counts in stochastic simulations. For each combination of cross-protection, enhancement, and R_0 pair, the average ratio (over 100 simulations) between cumulative DENV cases over 1 year (**a**) and 2 years (**b**) after the introduction of ZIKV is plotted against the ratio of cumulative ZIKV cases with and without immune-mediated interactions with DENV. Values above 1 indicate increases in counts and values below 1 indicated decreases compared to the average value from the corresponding scenario without enhancement or ZIKV cross-protection against DENV. For each enhancement and cross-protection scenario pair, linear model fits to the averages (over the 5 R_0 pairs considered) are displayed as gray lines. Negative slopes are consistent with the hypothesis that higher ZIKV incidence is associated with lower DENV incidence. For the case when DENV $R_0 = 2$ and ZIKV $R_0 = 2$, panel **c** shows the relative changes in cumulative DENV incidence over 2 years post-ZIKV introduction (blue points) and changes in DENV peak size (red points). Black vertical lines separate the enhancement scenarios and the level of cross-protection increases from left to right within these sections. Relative changes in peak size are based on the highest DENV incidence in 20 years post-ZIKV introduction versus 20 years pre-ZIKV.

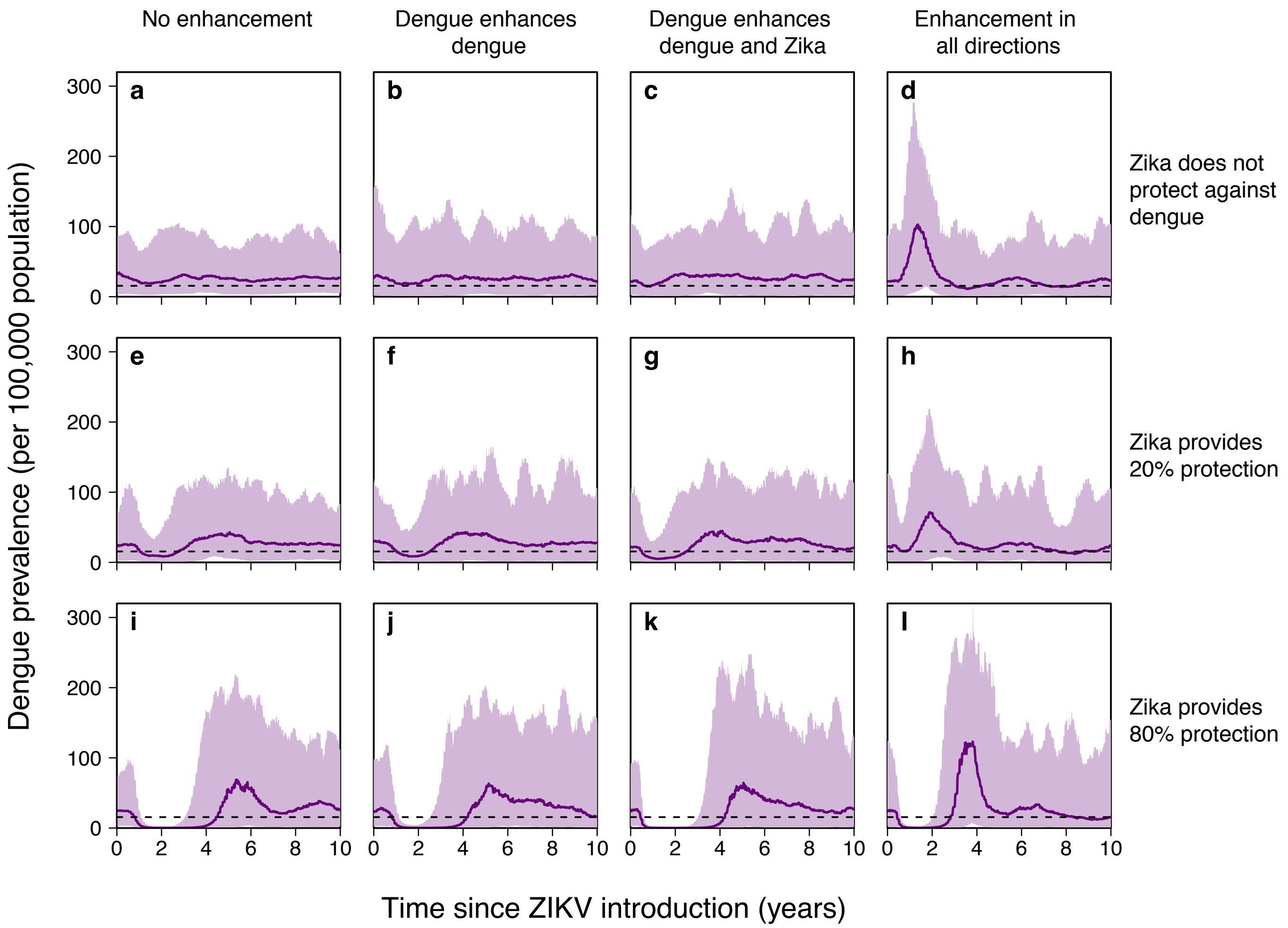

Supplementary Figure 11: Simulation results incorporating immune-mediated interactions between DENV and ZIKV. Mean and 95% inter-quantile range from stochastic simulations spanning 10 years post ZIKV-introduction. 100 simulations per scenario (see Methods for further details). ZIKV introduced 40 years after DENV is introduced in a susceptible population. Mosquitos infected with each of the four DENV serotypes are introduced as a Poisson process with an average of three per year. DENV and ZIKV reproduction numbers are 4 and 2 respectively. The dashed line indicates one-half of the average incidence in panel **a** which we use to define the start and end of DENV prevalence troughs. **a-d** Individuals with previous dengue exposure experience 20% of the DENV force of infection (FOI) that a fully susceptible person would. **e-h** Individuals with previous ZIKV exposure experience 80% of the FOI that a fully susceptible person would. **i-l** Individuals with ZIKV exposure experience 20% of the DENV FOI (same amount of cross-protection between dengue and Zika than between dengue serotypes).

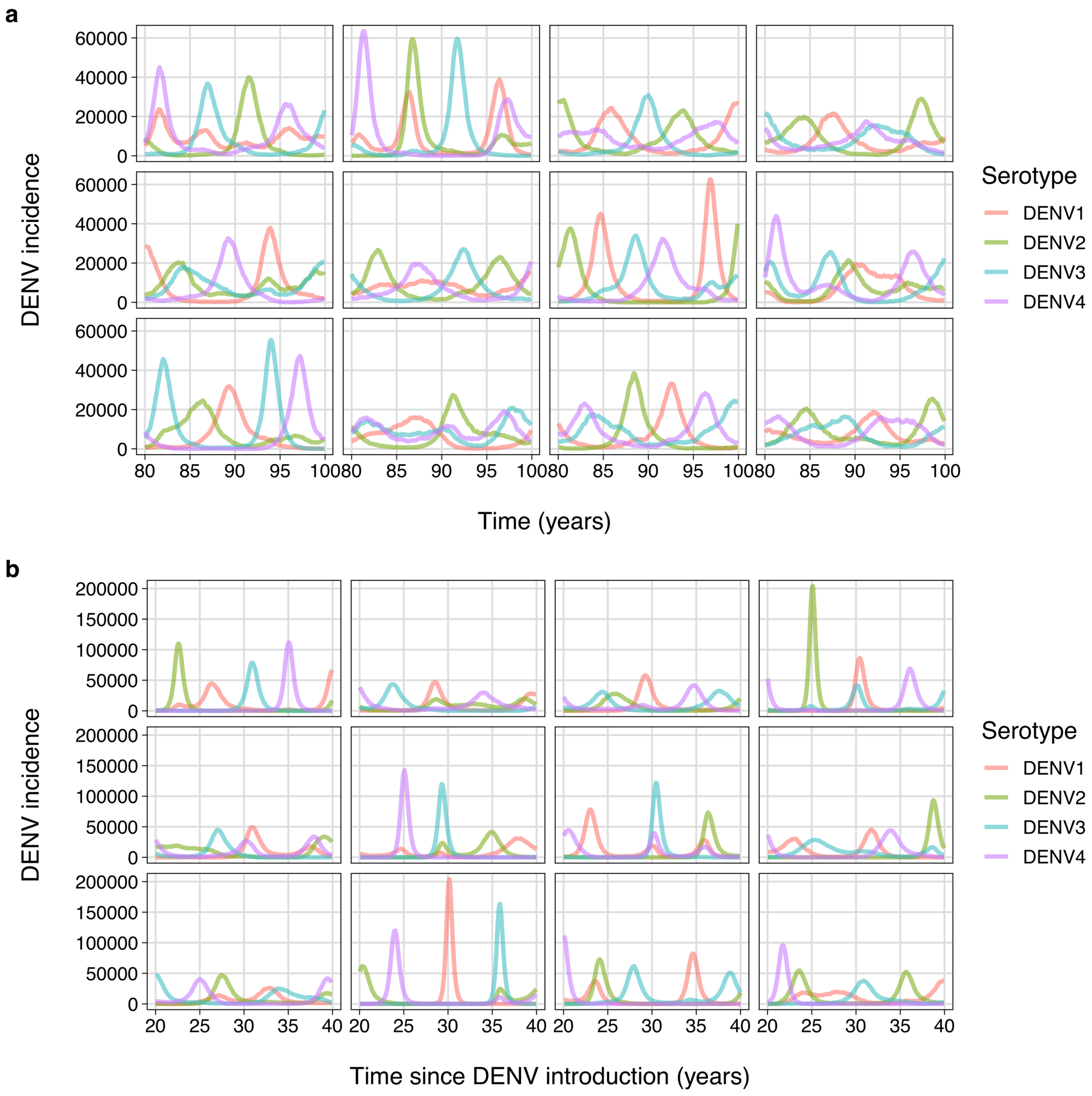

Supplementary Figure 12: Serotype-specific DENV incidence dynamics 20 years prior to the introduction of ZIKV. **(a)** ZIKV is introduced when DENV is in a stable state (time = 100). **(b)** ZIKV introduced 40 years after DENV is introduced in a susceptible population. Mosquitos infected with each of the four DENV serotypes are introduced as a Poisson process with an average of three per year. DENV and ZIKV reproduction numbers are 4 and 2 respectively.

State of DENV when ZIKV introduced	Cross-protection	Enhancement	Metric	Mean (95% IQR)
stable state	no cross-protection	No enhancement	Peak size	59.1 (39.8, 92.8)
40 years post introduction	no cross-protection	No enhancement	Peak size	97.0 (57.0, 170.0)
stable state	cross-protection (low)	No enhancement	Peak size	77.7 (52.9, 112.9)
40 years post introduction	cross-protection (low)	No enhancement	Peak size	110.5 (60.1, 206.1)
stable state	cross-protection (high)	No enhancement	Peak size	158.0 (120.3, 210.6)
40 years post introduction	cross-protection (high)	No enhancement	Peak size	178.2 (126.3, 262.6)
stable state	no cross-protection	No enhancement	Peak increase relative to baseline without cross-protection	1.0 (-)
40 years post introduction	no cross-protection	No enhancement	Peak increase relative to baseline without cross-protection	1.0 (-)
stable state	cross-protection (low)	No enhancement	Peak increase relative to baseline without cross-protection	1.3 (-)
40 years post introduction	cross-protection (low)	No enhancement	Peak increase relative to baseline without cross-protection	1.1 (-)
stable state	cross-protection (high)	No enhancement	Peak increase relative to baseline without cross-protection	2.7 (-)
40 years post introduction	cross-protection (high)	No enhancement	Peak increase relative to baseline without cross-protection	1.8 (-)
stable state	no cross-protection	No enhancement	Trough duration	0.3 (0.0, 2.3)
40 years post introduction	no cross-protection	No enhancement	Trough duration	1.4 (0.0, 3.6)
stable state	cross-protection (low)	No enhancement	Trough duration	1.0 (0.0, 2.6)
40 years post introduction	cross-protection (low)	No enhancement	Trough duration	1.9 (0.0, 3.9)
stable state	cross-protection (high)	No enhancement	Trough duration	3.4 (0.0, 4.4)
40 years post introduction	cross-protection (high)	No enhancement	Trough duration	3.5 (0.1, 5.3)
stable state	no cross-protection	DENV enhances DENV	Peak size	83.8 (54.0, 126.1)
40 years post introduction	no cross-protection	DENV enhances DENV	Peak size	122.7 (57.7, 220.7)
stable state	cross-protection (low)	DENV enhances DENV	Peak size	93.1 (61.4, 139.1)
40 years post introduction	cross-protection (low)	DENV enhances DENV	Peak size	135.4 (76.9, 232.5)
stable state	cross-protection (high)	DENV enhances DENV	Peak size	152.1 (113.9, 207.0)
40 years post introduction	cross-protection (high)	DENV enhances DENV	Peak size	180.5 (117.2, 286.0)
stable state	no cross-protection	DENV enhances DENV	Trough duration	0.7 (0.0, 2.2)
40 years post introduction	no cross-protection	DENV enhances DENV	Trough duration	1.4 (0.0, 3.9)
stable state	cross-protection (low)	DENV enhances DENV	Trough duration	1.3 (0.0, 2.8)
40 years post introduction	cross-protection (low)	DENV enhances DENV	Trough duration	1.9 (0.0, 4.0)
stable state	cross-protection (high)	DENV enhances DENV	Trough duration	3.1 (0.0, 4.4)
40 years post introduction	cross-protection (high)	DENV enhances DENV	Trough duration	3.2 (0.2, 5.5)
stable state	no cross-protection	DENV enhances DENV and ZIKV	Peak size	86.2 (53.3, 122.0)
40 years post introduction	no cross-protection	DENV enhances DENV and ZIKV	Peak size	128.3 (61.5, 299.6)
stable state	cross-protection (low)	DENV enhances DENV and ZIKV	Peak size	98.0 (72.6, 137.9)
40 years post introduction	cross-protection (low)	DENV enhances DENV and ZIKV	Peak size	138.5 (76.6, 248.3)
stable state	cross-protection (high)	DENV enhances DENV and ZIKV	Peak size	166.6 (131.0, 224.7)
40 years post introduction	cross-protection (high)	DENV enhances DENV and ZIKV	Peak size	199.2 (140.7, 288.8)
stable state	no cross-protection	DENV enhances DENV and ZIKV	Trough duration	0.8 (0.0, 2.4)
40 years post introduction	no cross-protection	DENV enhances DENV and ZIKV	Trough duration	1.4 (0.0, 3.5)
stable state	cross-protection (low)	DENV enhances DENV and ZIKV	Trough duration	1.5 (0.0, 3.0)
40 years post introduction	cross-protection (low)	DENV enhances DENV and ZIKV	Trough duration	2.2 (0.1, 4.8)
stable state	cross-protection (high)	DENV enhances DENV and ZIKV	Trough duration	3.5 (0.1, 4.4)
40 years post introduction	cross-protection (high)	DENV enhances DENV and ZIKV	Trough duration	3.6 (0.1, 5.4)
stable state	no cross-protection	Enhancement in all directions	Peak size	128.5 (76.0, 216.3)
40 years post introduction	no cross-protection	Enhancement in all directions	Peak size	163.1 (76.8, 282.1)
stable state	cross-protection (low)	Enhancement in all directions	Peak size	110.9 (67.6, 175.1)
40 years post introduction	cross-protection (low)	Enhancement in all directions	Peak size	152.5 (76.6, 247.2)
stable state	cross-protection (high)	Enhancement in all directions	Peak size	212.0 (153.0, 262.5)
40 years post introduction	cross-protection (high)	Enhancement in all directions	Peak size	229.9 (154.5, 375.6)
stable state	no cross-protection	Enhancement in all directions	Trough duration	1.2 (0.0, 3.1)
40 years post introduction	no cross-protection	Enhancement in all directions	Trough duration	1.3 (0.1, 3.5)
stable state	cross-protection (low)	Enhancement in all directions	Trough duration	0.9 (0.0, 2.8)
40 years post introduction	cross-protection (low)	Enhancement in all directions	Trough duration	1.3 (0.0, 3.7)
stable state	cross-protection (high)	Enhancement in all directions	Trough duration	2.2 (0.1, 3.1)
40 years post introduction	cross-protection (high)	Enhancement in all directions	Trough duration	2.3 (0.0, 3.8)

Supplementary Table 1: Peak and trough metrics of the stochastic compartmental model simulation results. The first column indicates the DENV immunity status of the population when ZIKV is introduced. For the stable state case, ZIKV was introduced after 100 years of stochastic four serotype DENV dynamics with initial conditions based on the steady state of the corresponding deterministic system. For the other simulations, ZIKV is introduced 40 years after the introduction of DENV with the population fully susceptible to DENV at time zero. See Supplementary Fig. 11 and Methods for details. Mean and 95% inter-quantile range (IQR) are displayed for peak size and trough duration, averaging over 100 simulations performed for each cross-protection and enhancement scenario. Peak increase is defined as the increase in peak DENV prevalence following ZIKV introduction relative to the baseline case without enhancement and without DENV-ZIKV cross-protection (all simulations incorporate cross-protection between DENV serotypes). Peak size is the largest DENV prevalence value (units of individuals per 100,000 population) during the 20 years following ZIKV introduction. Trough duration is defined as the number of years that DENV prevalence drops below an arbitrarily chosen factor of one half the mean DENV prevalence from 100 simulations with no enhancement and no DENV-ZIKV cross-protection for 20 years following ZIKV introduction. Our implementation of cross-protection and enhancement is described in the Methods.
