# Supplementary material for: Impacts of Zika emergence in Latin America on endemic dengue transmission
Source: Nat Commun. 2019 Dec 16;10:5730. doi: 10.1038/s41467-019-13628-x (PMC6915707; doi:10.1038/s41467-019-13628-x)
Supplement: Supplementary file 3 — Reporting Summary [file 41467_2019_13628_MOESM3_ESM.pdf]

nature research

Corresponding author(s): Rebecca K. Borchering and Derek A.T. Cummings

Last updated by author(s): Nov 1, 2019

Reporting Summary

Nature Research wishes to improve the reproducibility of the work that we publish. This form provides structure for consistency and transparency in reporting. For further information on Nature Research policies, see Authors & Referees and the Editorial Policy Checklist.

Statistics

For all statistical analyses, confirm that the following items are present in the figure legend, table legend, main text, or Methods section.

n/a	Confirmed	
☐	☒	The exact sample size (*n*) for each experimental group/condition, given as a discrete number and unit of measurement
☐	☒	A statement on whether measurements were taken from distinct samples or whether the same sample was measured repeatedly
☐	☒	The statistical test(s) used AND whether they are one- or two-sided<br>*Only common tests should be described solely by name; describe more complex techniques in the Methods section.*
☐	☒	A description of all covariates tested
☐	☒	A description of any assumptions or corrections, such as tests of normality and adjustment for multiple comparisons
☐	☒	A full description of the statistical parameters including central tendency (e.g. means) or other basic estimates (e.g. regression coefficient) AND variation (e.g. standard deviation) or associated estimates of uncertainty (e.g. confidence intervals)
☐	☒	For null hypothesis testing, the test statistic (e.g. *F*, *t*, *r*) with confidence intervals, effect sizes, degrees of freedom and *P* value noted<br>*Give P values as exact values whenever suitable.*
☐	☒	For Bayesian analysis, information on the choice of priors and Markov chain Monte Carlo settings
☐	☒	For hierarchical and complex designs, identification of the appropriate level for tests and full reporting of outcomes
☐	☒	Estimates of effect sizes (e.g. Cohen's *d*, Pearson's *r*), indicating how they were calculated

Our web collection on statistics for biologists contains articles on many of the points above.

Software and code

Policy information about availability of computer code

Data collection	We used R (v 3.4.2) and Rstudio (v 1.1.383) to collate the case data used in this study. The code used is provided in the UF-IDD/dengue-Zika-chik_Americas GitHub repository (DOI: 10.5281/zenodo.2566509).
Data analysis	We used R (v 3.4.2) and Rstudio (v 1.1.383) to analyze the data.

For manuscripts utilizing custom algorithms or software that are central to the research but not yet described in published literature, software must be made available to editors/reviewers. We strongly encourage code deposition in a community repository (e.g. GitHub). See the Nature Research guidelines for submitting code & software for further information.

Data

Policy information about availability of data

All manuscripts must include a data availability statement. This statement should provide the following information, where applicable:
- Accession codes, unique identifiers, or web links for publicly available datasets
- A list of figures that have associated raw data
- A description of any restrictions on data availability

The case count and population data gathered and analyzed in this study are available in GitHub repository UF-IDD/dengue-Zika-chik_Americas (DOI: 10.5281/zenodo.2566509). This repository also includes a detailed description of the data collation procedure including the web links for the publicly available datasets we utilized.

Field-specific reporting

Please select the one below that is the best fit for your research. If you are not sure, read the appropriate sections before making your selection.

☐ Life sciences ☐ Behavioural & social sciences ☒ Ecological, evolutionary & environmental sciences

For a reference copy of the document with all sections, see nature.com/documents/nr-reporting-summary-flat.pdf

Ecological, evolutionary & environmental sciences study design

All studies must disclose on these points even when the disclosure is negative.

Study description	We performed an observational study of passive surveillance data describing observed counts of dengue, Zika, and chikungunya cases in states and departments in Brazil and Colombia within the time interval (1999-2017). There was no assignment to treatment.
Research sample	Probable dengue, Zika, and chikungunya cases from passive surveillance data. Age, gender, and sex were not specified in the publicly available data we utilized. The data includes cases from all groups.
Sampling strategy	This was an observational study and we used the data available. No sampling was performed.
Data collection	Case data was collected by the Ministerio da Saude Brasil and Instituto Nacional de Salud Colombia. We accessed publicly available data from websites and also digitized case data published in Epidemiological Bulletins. Population data was collected by Instituto Brasileiro de Geografia e Estatística and Departamento Administrativo Nacional de Estadística Colombia.
Timing and spatial scale	The study includes probable cases occurring between 1999 and 2017 within Brazil and Colombia.
Data exclusions	No data were excluded.
Reproducibility	This was an observational study and no experimental treatment was used. For Bayesian statistical models we fit multiple chains and report convergence criteria.
Randomization	This was an observational study and we did not make any assignments to groups.
Blinding	No experimental treatment was delivered. Analysts collated data retrospectively and had access to all available data.

Did the study involve field work? ☐ Yes ☒ No

Reporting for specific materials, systems and methods

We require information from authors about some types of materials, experimental systems and methods used in many studies. Here, indicate whether each material, system or method listed is relevant to your study. If you are not sure if a list item applies to your research, read the appropriate section before selecting a response.

Materials & experimental systems

n/a	Involved in the study	
☒	☐	Antibodies
☒	☐	Eukaryotic cell lines
☒	☐	Palaeontology
☒	☐	Animals and other organisms
☒	☐	Human research participants
☒	☐	Clinical data

Methods

n/a	Involved in the study	
☒	☐	ChIP-seq
☒	☐	Flow cytometry
☒	☐	MRI-based neuroimaging
